# Familial hypercholesterolaemia in South Africans: tracking findings and developments over time

**Published:** 2009-02

**Authors:** Rhena Delport

**Affiliations:** Department of Chemical Pathology/Department of Family Medicine, School of Medicine, Faculty of Health Sciences, University of Pretoria, Pretoria

## Abstract

**Summary:**

This review discusses the 1987 article by Wyndham, Seftel, Pilcher and Baker on familial hypercholesterolaemia (FH) and myocardial infarction (MI) in young Afrikaners, in terms of the significance at the time of publication, as well as the relevance of their findings versus current observations on hypercholesterolaemia in South Africa. Risk factors for coronary heart disease (CHD) were investigated in this study, with specific reference to familial hypercholesterolaemia. The significance of Wyndham’s article is evaluated with regard to the contributions on hypercholesterolaemia by other South African researchers that preceded this publication. The clinical diagnostic criteria that were applied to identify possible FH at the time of publication are compared with currently employed criteria and guidelines. This review also acknowledges and honours other clinicians and researchers who, like Wyndham *et al.*, have made significant contributions to the diagnosis and treatment of FH in South Africans.

## Summary

The low-density lipoprotein receptor was discovered in 1974 by Brown and Goldstein,^1^ who demonstrated that FH was caused by a defect in the receptor.^2^ FH was characterised as a common autosomal dominant trait occurring in approximately one in 500 persons in the general population.^3^ Goldstein and Brown distinguished between hetero- and homozygote plasma levels of low-density lipoprotein cholesterol, and discussed the onset of xanthomata, disease progression and life expectancy in a follow-up publication.^3^ The contribution from these authors in 1978 describes the three cardinal features of familial hypercholesterolemia (FH) as:

● ‘a selective elevation in the plasma level of one cholesterolcarrying lipoprotein, low density lipoprotein (LDL);
● a selective deposition of LDL-derived cholesterol in macrophage-like scavenger cells throughout the body, but not in parenchymal cells; and
● inheritance as an autosomal dominant trait with gene dosage effect, i.e., the disease is more serious in patients with the homozygous than with the heterozygous state.’^2^

In a recent review on gene variants affecting plasma LDL cholesterol, 4 Burnett and Hooper refer to Goldstein, Hobbs and Brown’s main findings on FH.^5^ Goldstein and co-authors estimated the frequency of FH among Caucasians as 1:500, and reported a prevalence of 1:100 in Afrikaners. They found that plasma LDL cholesterol concentrations were typically elevated to two to three times normal values, and approximately 75% of male heterozygotes and 45% of female heterozygotes developed CHD by the age of 60 years.^5^ In homozygous FH, found in one in one million people, plasma LDL cholesterol concentrations were extremely high (in the order of 15–24 mmol/l) and atherosclerotic CHD developed in early childhood.5 Approximately 45% of male and 20% of female patients were predicted to develop coronary heart disease by the age of 50.^5^

This review takes an in-depth look at FH in South Africans, using the publication of Wyndham *et al.*^6^ as a reference point to explore changes over time in the risk profile of FH subjects for cardiovascular disease. Developments in clinical diagnosis and risk assessment are also discussed and the significance of FH in the bigger picture of prevalence of cardiovascular disease in South Africans is scrutinised.

## Significance of the article by Wyndham *et al.*

Although difficult to ascertain with certainty, CHD was commonly observed in South Africans around the 1970s, as is evident from the publication of Wyndham in 1969, ‘The problem of coronary heart disease with special reference to the influence of physical activity’^7^ and Stein (1977), ‘The Lipid Disorders Centre at the Transvaal Memorial Hospital for Children: A review of the first 30 months’.^8^ In 1978, Wyndham reported that the 1970 mortality rates for ischaemic heart disease (IHD) in white South Africans exceeded the rates in the USA and Finland, countries with known high IHD mortality rates.^9^ This was particularly evident in the younger age groups.^9^ A number of reports on FH in South Africans followed. Seftel *et al.*^10^ in 1980 calculated the prevalence of homozygous and heterozygous FH as one in 30 000 and one in 100, respectively, while Pedoe^11^ reported a prevalence in the same year of 1:75 in white Afrikaners, and an overall incidence in other South Africans of approximately 1:500. Table 1 summarises data on the incidence of FH in South Africans from 1980 to 1987 as well as in the following years.

**Table 1:** 

*Year*	*Author*	*Population group*	*Homozygote incidence*	*Heterozygote incidence*	*FH (unspecified)*
1980	Seftel *et al.*^10^	Total population of Afrikaners aged < 50 years within 150 km of Johannesburg in 1979, reported as 951 000	1:30 000	1:100	
1980	Pedoe11	Afrikaners, Other South Africans		1:75 1:500	
1986	Jooste *et al.*^12^	Predominantly Afrikaner communities in the south-western Cape			1:87
1987	Seftel, Wyndham and Dos Santos^13^	Coronary bypass surgery patients with severe hypercholesterolaemia			1:4
1989	Seftel *et al.*^14^	Representative sample of 403 young Jewish men resident in Johannesburg			1:67 of the total sample
1996	Steyn *et al.*^15^	1 612 randomly selected participants from a rural Afrikaner community			1.4 (95% CI: 0.91, 2.1) (~1/72 individuals)
1998	Steyn *et al.*^16^	12 842 patients seen by the 200 general practitioners			3.1%

During the period between 1980 and 1987, indications for a diagnosis of FH were defined by Seftel *et al.*^10^ as a serum total cholesterol level of greater than14.3 mmol/l and xanthomata in first decade of life. Pedoe^11^ diagnosed FH when the following was observed:

● a total cholesterol level above certain age and specific cut-off points
● the presence of tendon xanthomata, and
● similar findings in a relative, especially parents.

A family history of early CHD was also regarded as a clue.^11^

Part of the significance of the article by Wyndham *et al.*^6^ relates to the detailed nature of and systematic approach to the identification of FH subjects in the study. The criteria applied by Wyndham *et al.* for the diagnosis of FH were:

● a serum cholesterol level of equal to or greater than 7.0 mmol/l plus tendon xanthomata; or
● a serum cholesterol level of equal to or greater than 8.5 mmol/l without tendon xanthomata plus, in at least one first-degree relative, serum cholesterol level of equal to or greater than 7.0 mmol/l with tendon xanthomata or a serum cholesterol level of equal to or greater than 8.5 mmol/l without tendon xanthomata,
● a juvenile or child first-degree relative, a serum cholesterol level of equal to or greater than 7.0 mmol/l without peripheral signs was accepted.

Clinical diagnoses of FH are currently commonly guided by the Dutch Lipid Network criteria and UK-based Simon Broome Register Group criteria.

## The Dutch Lipid Clinic Network criteria

The Dutch Lipid Clinic Network criteria^17^ assess the likelihood of FH using a scoring system which takes into account the subject’s history of premature coronary artery disease and/or cerebral or peripheral vascular disease, presence of tendon xanthomata and arcus corneae, LDL cholesterol concentration, the subject’s family history, and presence of a mutation in the LDL receptor gene. Subjects can be classified as having definite, probable, possible, unlikely, or indeterminate (insufficient evidence available) FH.

## The Simon Broome criteria

According to the Simon Broome Register Group,^18^ a definite diagnosis of FH requires:

1. Total cholesterol level above 7.5 mmol/l in adults or a total cholesterol level above 6.7 mmol/l for children under 16, (either pre-treatment or highest on treatment)^19^

OR

LDL levels above 4.9 mmol/l in adults (4.0 mmol/l in children) (either pre-treatment or highest on treatment)

PLUS

2. Tendon xanthomata in patient or relative (parent, child, sibling, grandparent, aunt, uncle)

OR

3. DNA-based evidence of an LDL receptor mutation or familial defective apo B-100 or a PCSK9 mutation.

Possible FH is defined as (1) above plus one of (4) or (5):

4. Family history of MI before age 50 in grandparent, aunt, uncle or before age 60 in parent, sibling or child.

5. Family history of raised total cholesterol: greater than 7.5 mmol/l in adult first- or second-degree relative or greater than 6.7 mmol/l in child, brother or sister aged younger than 16 years.

## The NICE clinical guidelines

The National Institute for Health and Clinical Excellence clinical guidelines and evidence review for familial hypercholesterolaemia: the identification and management of adults and children with familial hypercholesterolaemia (NICE clinical guideline 71),^20^ published in August this year, have provided immensely valuable detailed guidelines. Key priorities for implementation are set for diagnosis and identification of people with FH, using cascade testing, and are quoted below.

## Key priorities for implementation

## Diagnosis

● A family history of premature coronary heart disease should always be assessed in a person being considered for a diagnosis of FH.
● In children at risk of FH because of one affected parent, the following diagnostic tests should be carried out by the age of 10 years or at the earliest opportunity thereafter
− A DNA test if the family mutation is known
− LDL-C concentration measurement if the family mutation is not known. When excluding a diagnosis of FH, a further LDL-C measurement should be repeated after puberty because LDL-C concentrations change during puberty.
● Coronary heart disease risk estimation tools such as those based on the Framingham algorithm should not be used because people with FH are already at a high risk of premature coronary heart disease.

## Identifying people with FH using cascade testing

● Healthcare professionals should offer all people with FH a referral to a specialist with expertise in FH for confirmation of diagnosis and initiation of cascade testing.
● Cascade testing using a combination of DNA testing and LDL-C concentration measurement is recommended to identify affected relatives of those index individuals with a clinical diagnosis of FH. This should include at least the first- and second- and, when possible, third-degree biological relatives.
● The use of a nationwide, family-based follow-up system is recommended to enable comprehensive identification of people affected by FH.’

Based on the best available evidence, guidelines concerning diagnosis of FH and identifying people with FH using cascade testing are quoted below.

## Guidelines

## 1.1 Diagnosis

1.1.1 ‘Healthcare professionals should consider the possibility of FH in adults with raised cholesterol (total cholesterol typically greater than 7.5 mmol/l), especially when there is a personal or a family history of premature coronary heart disease.
1.1.2 Healthcare professionals should exclude secondary causes of hypercholesterolaemia before a diagnosis of FH is considered.
1.1.3 A diagnosis of FH should be made using the Simon Broome criteria, which include a combination of family history, clinical signs (specifically tendon xanthomata), cholesterol concentration and DNA testing.
1.1.4 Healthcare professionals should inform people with a diagnosis of FH based on the Simon Broome criteria that they have a clinical diagnosis of FH.
1.1.5 Healthcare professionals should consider a clinical diagnosis of homozygous FH in adults with an LDL-C concentration greater than 13 mmol/l and in children/young people with an LDL-C concentration greater than 11 mmol/l. All people with a clinical diagnosis of homozygous FH should be offered referral to a specialist centre.
1.1.6 To confirm a diagnosis of FH, healthcare professionals should undertake two measurements of LDL-C concentration because biological and analytical variability occurs.
1.1.7 Healthcare professionals should be aware that the absence of clinical signs (for example, tendon xanthomata) in adults and children/young people does not exclude a diagnosis of FH.
1.1.8 A family history of premature coronary heart disease should always be assessed in a person being considered for a diagnosis of FH (see Simon Broome criteria, appendix E).
1.1.9 When considering a diagnosis of FH, healthcare professionals with expertise in FH should use standardised pedigree terminology to document, when possible, at least a three-generation pedigree. This should include relatives’ age of onset of coronary heart disease, lipid concentrations and smoking history. For deceased relatives, the age and cause of death, and smoking history should be documented. If possible, the index individual should verify this information with other family members.
1.1.10 Ultrasonography of the Achilles tendon is not recommended in the diagnosis of FH.
1.1.11 Coronary heart disease risk estimation tools such as those based on the Framingham algorithm should not be used because people with FH are already at a high risk of premature coronary heart disease.
1.1.12 Healthcare professionals should offer people with a clinical diagnosis of FH a DNA test to increase the certainty of their diagnosis and to aid diagnosis among their relatives.
1.1.13 Healthcare professionals should inform all people who have an identified mutation diagnostic of FH that they have an unequivocal diagnosis of FH even if their LDL-C concentration does not meet the diagnostic criteria (see appendix E).
1.1.14 In a family where a DNA mutation is identified, not all family members may have inherited the mutation. When DNA testing has excluded FH in a member of a family, healthcare professionals should manage the person’s coronary heart disease risk as in the general population. [Lipid modification: cardiovascular risk assessment and the modification of blood lipids for the primary and secondary prevention of cardiovascular disease (NICE clinical guideline 67).]
1.1.15 In children at risk of FH because of one affected parent, the following diagnostic tests should be carried out by the age of 10 years or at the earliest opportunity thereafter.
● A DNA test if the family mutation is known
● LDL-C concentration measurement if the family mutation is not known. When excluding a diagnosis of FH a further LDL-C measurement should be repeated after puberty because LDL-C concentrations change during puberty.
1.1.1 In children at risk of homozygous FH because of two affected parents or because of the presence of clinical signs, for example, cutaneous lipid deposits (xanthomata), LDL-C concentration should be measured before the age of 5 years or at the earliest opportunity thereafter. If the LDL-C concentration is greater than 11 mmol/l then a clinical diagnosis of homozygous FH should be considered.

## 1.2 Identifying people with FH using cascade testing

1.2.1 Healthcare professionals should use systematic methods (that is, cascade testing) for the identification of people with FH.
1.2.2 Healthcare professionals should offer all people with FH a referral to a specialist with expertise in FH for confirmation of diagnosis and initiation of cascade testing.
1.2.3 Healthcare professionals with expertise in FH should explain what is meant by cascade testing, and discuss its implications with all people with FH.
1.2.4 Cascade testing using a combination of DNA testing and LDL-C concentration measurement is recommended to identify affected relatives of those index individuals with a clinical diagnosis of FH. This should include at least the first- and second- and, when possible, third-degree biological relatives.
1.2.5 In families in which a mutation has been identified, the mutation and not LDL-C concentration should be used to identify affected relatives. This should include at least the first- and second- and, when possible, third-degree biological relatives.
1.2.6 In the absence of a DNA diagnosis, cascade testing using LDL-C concentration measurements should be undertaken to identify people with FH.
1.2.7 To diagnose FH in relatives of an index individual, the gender- and age-specific criteria for LDL-C concentration in appendix E should be used. The Simon Broome LDL-C criteria for index individuals should not be used because this will result in under diagnosis.
1.2.8 The use of a nationwide, family-based follow-up system is recommended to enable comprehensive identification of people affected by FH.
1.2.9 Healthcare professionals should be aware of the latest guidance on data protection when undertaking cascade testing.’

The criteria and cut-off levels used by Wyndham *et al.*^6^ were in actual fact more stringent than those applied within the diagnostic criteria for index individuals (Simon Broome criteria).^18^ Subjects with a serum cholesterol level of equal to or greater than 7.0 mmol/l were classified by Wyndham as possible FH, whereas the Simon Broome Register Group regarded greater than 7.5 mmol/l in the presence of tendon xanthomata as indicative of FH. One obviously has to consider differences in methodology and reference ranges, as well as changes in precision of methodology in cholesterol determination over time when comparing cut-off levels.

Currently, the within-subject and between-subject coefficient of variation values of total serum cholesterol are estimated at 5.4 and 15.2%, respectively, and desirable analytical quality specifications for imprecision, bias and total error of total serum cholesterol are 2.7, 4.0 and 8.5%, respectively.^21^ When the 0.5-mmol/l difference between the cut-off used by Wyndham *et al.* and that set within the Simon Broom criteria, expressed as a percentage of 7 mmol/l, a 0.7% difference is observed. When compared with the above-reported calculations for total cholesterol, the difference in cut-off levels appears clinically insignificant and would probably not affect the diagnosis of FH patients. The suggestion in the NICE guidelines^20^ that two measurements of LDL-C concentration be undertaken to confirm a diagnosis of FH because of biological and analytical variability (Guideline 1.1.16) is, however, significant and laudable.

Similarly, the child serum cholesterol level of equal to or greater than 7.0 mmol/l cut-off level is higher than the proposed > 6.7 mmol/l of the Simon Broome Register Group. This could possibly have contributed to false negative diagnoses of FH in children. False negatives may arise when tendon xanthomata do not accompany increased total cholesterol levels, and this has a profound effect on the diagnosis of FH. Both Wyndham *et al.* and the Simon Broome Register Group regarded the presence of tendon xanthomata as a prerequisite for the diagnosis of FH. The NICE diagnostic guideline that merits specific attention in this regard is that the absence of clinical signs of FH in adults and children/young people does not exclude a diagnosis of FH.

Furthermore, LDL cholesterol level was not included as a criterion by Wyndham *et al.*,^6^ nor was DNA-based evidence of an LDL receptor mutation. Methods employed for LDL determination were not as accurate in 1987 and would probably not have added value at that time. Although the laboratory methods had been established by South Africans during the period that preceded the publication of Wyndham *et al.*, this evidence of FH was not included as a criterion. These methods included LDL receptor activity^22^,^23^ tests and enzyme-restriction fragment-length polymorphisms of the low-density lipoprotein receptor gene.^24^-^26^

Missed diagnosis of FH is also of major concern, especially the diagnosis of FH in both primary and tertiary-level care, as reported by Bates *et al.*^27^ and Neil *et al.*^28^ In a recent review in the *European Heart Journal* (2008),^29^ Rees calculates that less than 20% of FH index cases are ascertained. In 1996, Hitzeroth^30^ reported FH to be very common in South Africa, with approximately 62 000 existing cases and 1 100 new cases being added to the pool each year. An extrapolation to the entire South African population during the same year^15^ suggests that there were about 112 000 FH patients in the country who were under-diagnosed as a group and therefore not receiving the care that would help to reduce the burden of FH-associated CHD in South Africa.

In an editorial,^31^ Marais very recently urged that ‘… much more needs to be done to fully characterise severe dyslipidaemias and translate therapeutic developments into practice.’ An increased focus on FH is observed nationally and internationally and hopefully this will drive ‘the delivery of systematic care for patients with FH and (to) establish a family cascade testing programme and a genetic register for the condition, as suggested by Rees.’^29^

## Relevance of Wyndham *et al.’s* findings on FH

Hypercholesterolaemia and smoking were identified as the two major risk factors for myocardial infarction in the South African population studied by Wyndham *et al.*,^6^ whereas hypertension, diabetes and hyperuricaemia did not appear to be implicated as risk factors. These findings differ somewhat from the results of a similar study published in 2004. Jansen *et al.*^32^ performed a retrospective, multicentre cohort study to assess which additional classical risk factors influence excess mortality in heterozygous FH. Patients (*n* = 2 400) from 27 Dutch lipid clinics were enrolled in the study when diagnosed with FH, based on either the presence of a low-density lipoprotein receptor mutation or strict clinical criteria. They found that male gender, smoking, hypertension, diabetes mellitus, low HDL cholesterol and high lipoprotein(a) levels were important risk factors for CVD in Dutch FH patients. The risk factors, however, only explained 18.7% of the variation in the occurrence of CVD in this patient group. Evidently there are other contributing factors to CHD.

## The bigger picture

With the added value provided by identification of new mutations, and acknowledgement of multiple risk factors, individuals with FH are probably relatively well catered for in South Africa, thanks to researchers like Wyndham *et al.* What is of great concern, however, is the increase in hypertension, diabetes and obesity in specifically black South Africans and the increased burden of disease.^33^,^34^ High cholesterol level is now recognised as an important cardiovascular risk factor in all population groups in South Africa.^35^ Mensah^34^ states that based on an estimate from the Global Burden of Disease project between 1996 and 2020,^36^ CHD deaths in sub-Saharan Africa will increase per annum by 125%, to 263 000 in men and by 141%, to 222 000 in women.

In the INTERHEART Africa study the population attributable risk for initial myocardial infarction (MI) is calculated as 89.2% with modelling of five risk factors (history of smoking, diabetes and hypertension, abdominal obesity, and ratio of apolipoprotein B to apolipoprotein A-1).^33^ The risk for MI appears to increase with higher income and education levels in the black African group, in contrast^33^ to findings in the other African groups studied in the overall INTERHEART group.^37^

To conclude, lifestyle modification and early diagnosis of CHD are of utmost importance, as stated very elegantly in a recent editorial by Mensah:^34^ ‘An aggressive approach that combines environmental, policy and legislative interventions for health promotion and primary prevention, coupled with improved access to evaluation, treatment and control of hypertension and other major risk factors, provides the best strategy for averting an epidemic of IHD in sub-Saharan Africa.’
